# Ventriculoperitoneal Shunt Malfunction Due to a Fibrous Capsule Obstructing the Peritoneal Catheter

**DOI:** 10.7759/cureus.6831

**Published:** 2020-01-31

**Authors:** Jonathan Heaney, Reuben Johnson

**Affiliations:** 1 Neurosurgery, Wellington Regional Hospital, Wellington, NZL; 2 Neurosurgery, Wellington Hospital, Wellington, NZL

**Keywords:** ventriculoperitoneal shunts, obstruction, fibrous capsule, peritoneal catheter

## Abstract

Ventriculoperitoneal shunt obstruction is a common neurosurgical condition. Obstruction of the peritoneal catheter is less common than other sites of blockage in the system. We present a case of a ventriculoperitoneal shunt blockage caused by a sheath of hyalinised fibrous tissue that had encased the distal side slits of the peritoneal catheter resulting in sub-total blockage. The patient made a complete recovery following the removal of this fibrous tissue from the distal peritoneal catheter without the need to revise the entire shunt.

## Introduction

Placement of ventriculoperitoneal shunts as a means of cerebrospinal fluid (CSF) diversion to treat hydrocephalus is a commonly performed operation in neurosurgery. A common complication is a subsequent blockage which usually presents with symptoms attributable to the recurrence of hydrocephalus. The ventricular catheter or valve is the most common site of blockage and can be caused by debris such as blood or excess protein in the CSF [1]. Additionally, ventricular catheter blockage may be caused by choroid plexus becoming entangled in and obstructing the perforations at the tip of the ventricular catheter. The blockage of the peritoneal catheter may be caused by knot formation, migration, pseudocyst formation, or the presence of side slits in a closed-ended peritoneal catheter [2-5]. Side slits were initially designed as a secondary safety valve should the open end of the peritoneal catheter be obstructed by abdominal contents. However, debris may accumulate in the dead space beneath the slits leading to obstruction, and many neurosurgeons now cut short the distal peritoneal catheter tip, thereby removing the portion of the peritoneal catheter containing side slits. We describe a case of peritoneal catheter obstruction at the site of these side slits by a fibrotic capsule. The blockage of the peritoneal catheter by a fibrous sheath is a rare phenomenon; initially, it was described in 1983, but it has only been described since by one author in two case reports [6-8].

## Case presentation

A 33-year-old male presented with an approximate four-day history of headache, nausea and vomiting, and decreased visual acuity. He had a right occipital ventriculoperitoneal shunt in situ (Medtronic Strata II Valve (Medtronic Inc, Minneapolis, USA), Performance setting 1.0) for communicating hydrocephalus diagnosed in childhood. The exact medical details from his childhood were not clear. His first shunt was inserted at two years of age. His last revision was 18 months prior when the valve had been replaced. His peritoneal catheter had been changed 10 years ago after it had migrated into pre-peritoneal fat. It was replaced with a Medtronic standard, 90 cm, Barium-impregnated closed-ended peritoneal catheter, with side slits.

**Figure 1:**
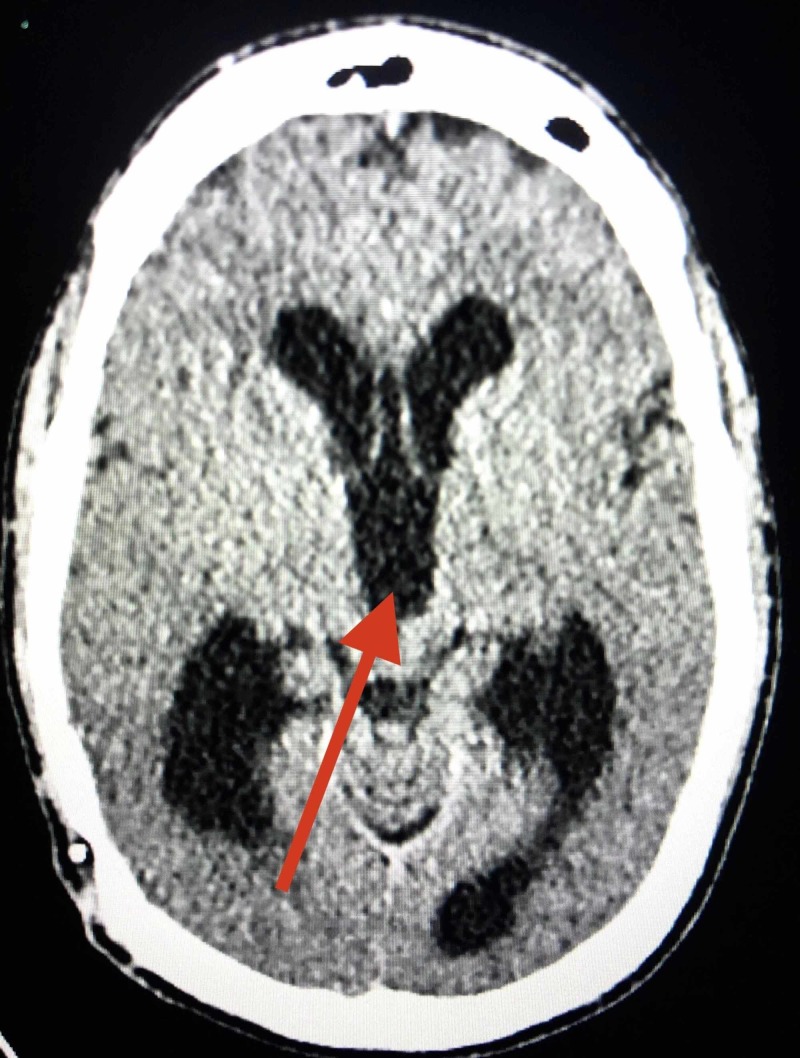
Pre-operative axial computed tomography of the head; arrow indicating dilation of third ventricle

He was afebrile and alert, and his abdomen was not tender. The valve reservoir felt normal to palpate and refilled briskly following pressure being applied to it. Blood tests, including inflammatory markers, were normal. Computed tomography (CT) head demonstrated hydrocephalus, and shunt series X-rays failed to demonstrate a fracture or disconnection in the shunt system (Figure 1). Decreasing the performance setting of his valve failed to improve his symptoms. Shunt obstruction was suspected and because of the duration of his symptoms, and since his valve reservoir refilled briskly after having pressure applied to it, this obstruction was suspected to be of the peritoneal catheter.

**Figure 2:**
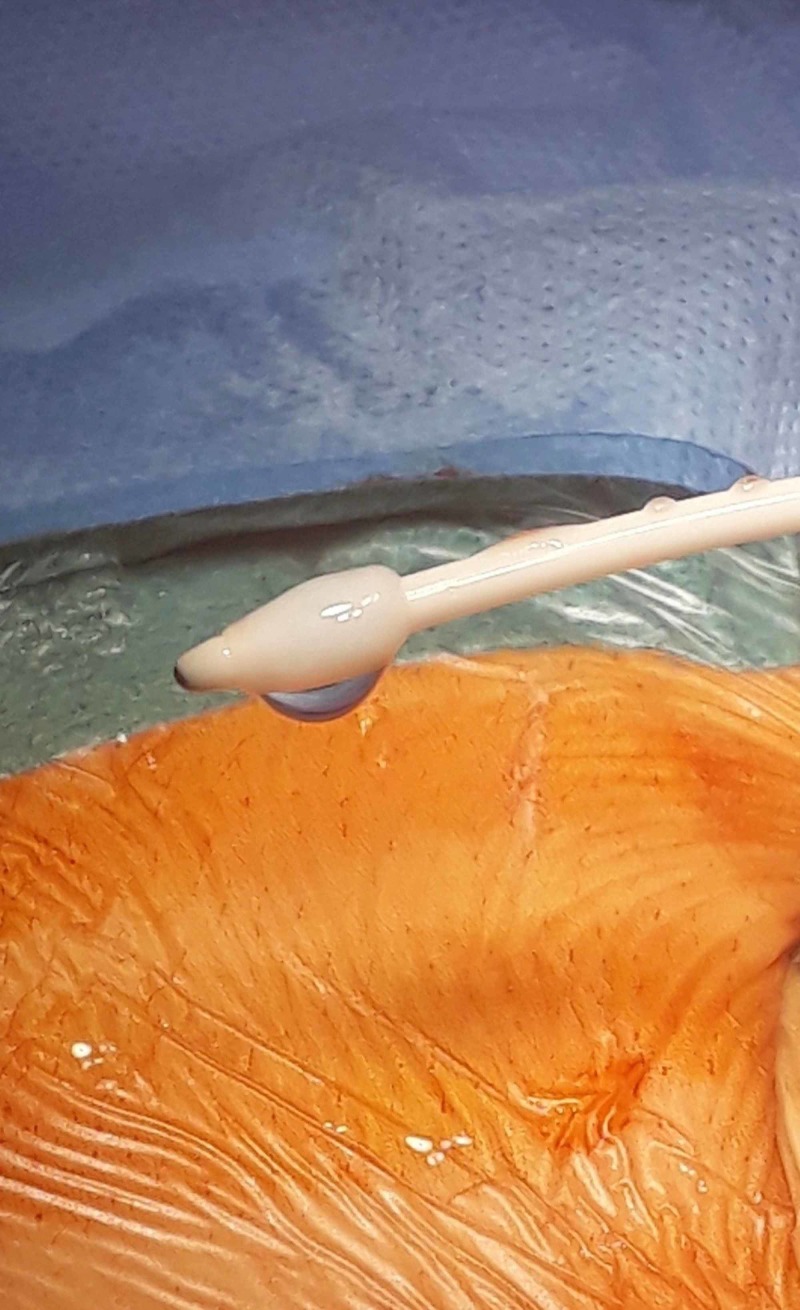
Peritoneal catheter with fibrous sheath encasing the side slits at the distal end of the catheter but not the tip itself

**Figure 3:**
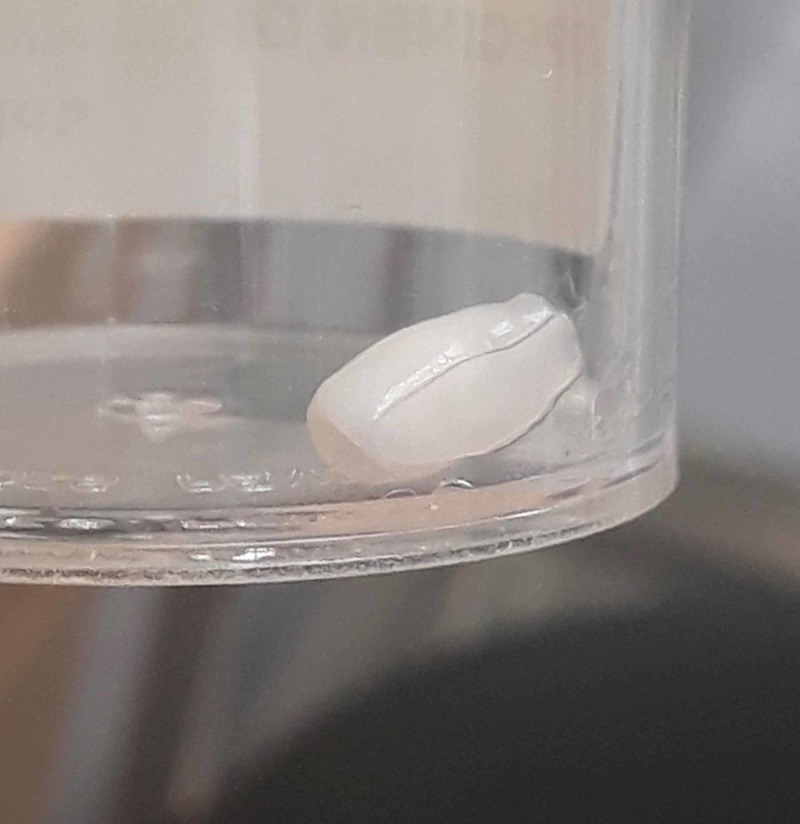
Fibrous capsule after removal

**Figure 4:**
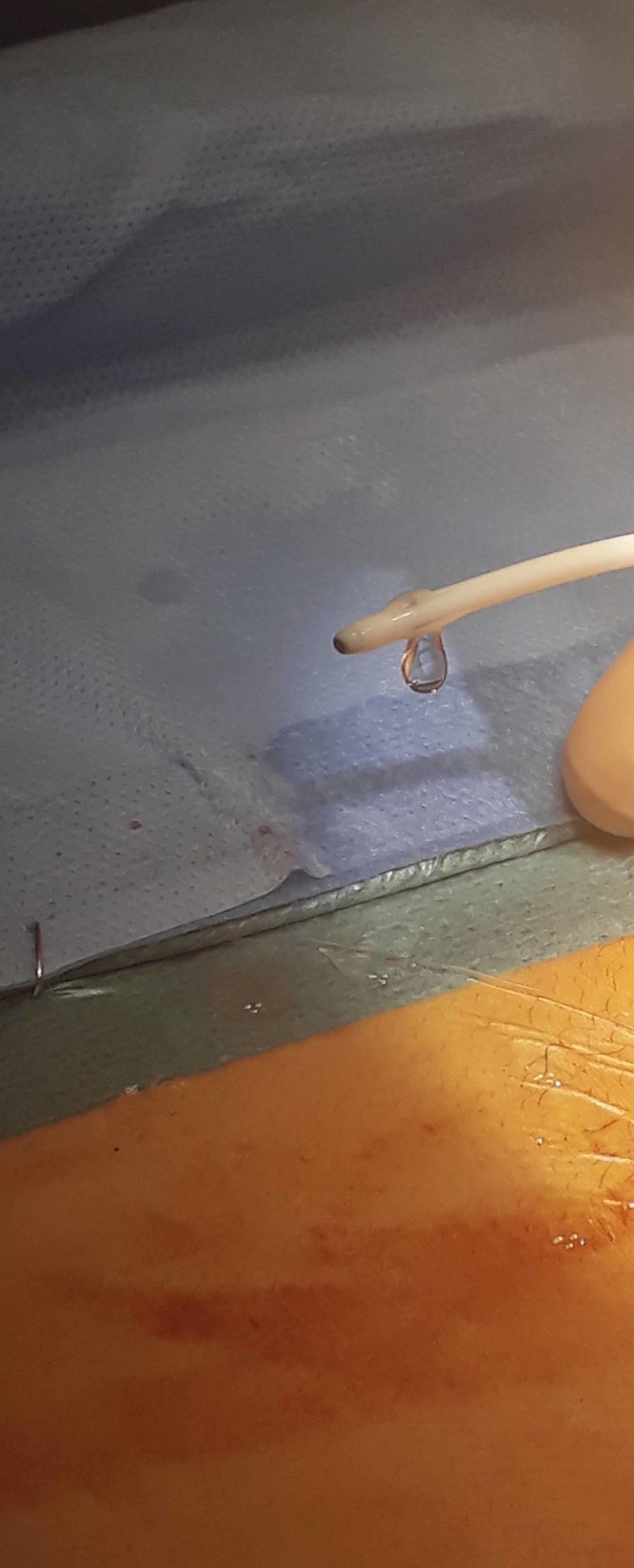
Distal catheter tip after removal of fibrous capsule, with patent slits visible

He was taken to the theatre where his previous abdominal incision was reopened and the peritoneal catheter located. During delivery of the peritoneal catheter from the peritoneal cavity, resistance was encountered at the peritoneum. The peritoneum was opened further to allow delivery of the distal tip of the peritoneal catheter. The distal end (around the side slits in the catheter, but not the tip itself), was found to be encased in a 1 cm long bulbous fibrotic sheath (Figures 2-3). Clear fluid (subsequently confirmed to be sterile CSF) was dripping from the catheter tip, albeit very slowly. Upon removal of this bulbous fibrotic sheath, CSF began to flow at a greater rate through the catheter (Figure 4). Additional perforations were cut into the side of the catheter, which was then placed back inside the peritoneal cavity. The cranial wound was not opened and as such, the ventricular catheter and valve were not exposed.

**Figure 5:**
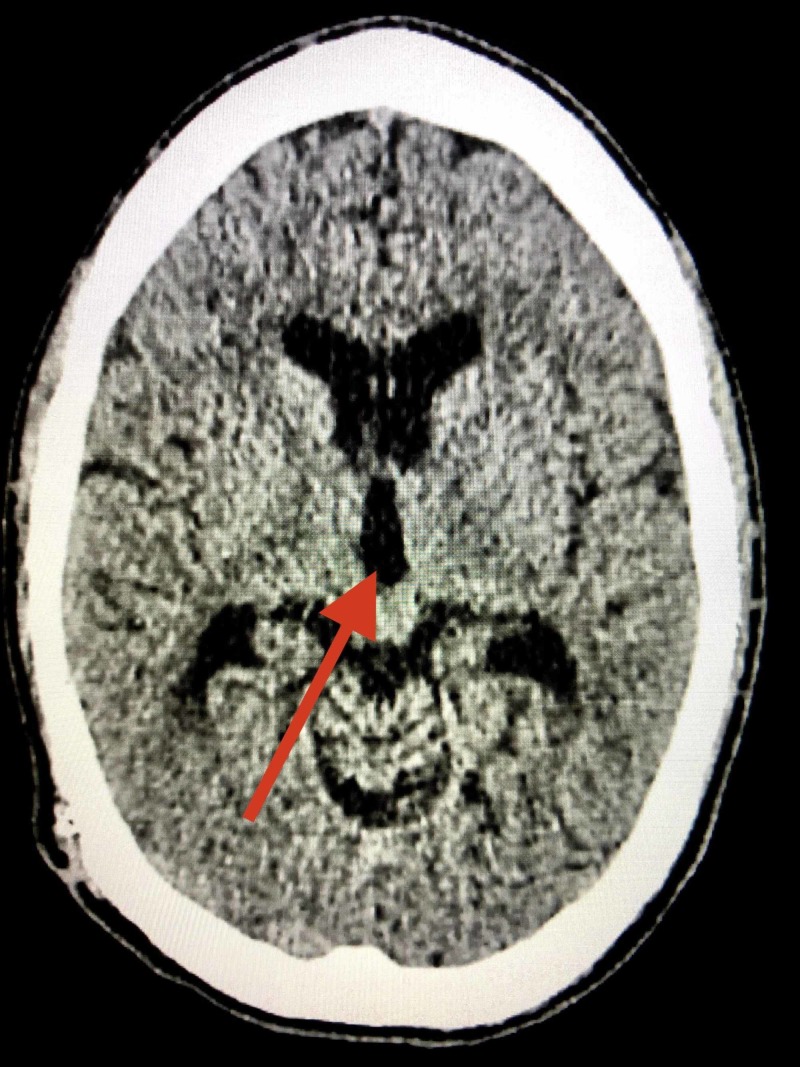
Post-operative axial computed tomography of the head demonstrating resolution of hydrocephalus; arrow indicating reduction in size of third ventricle

**Figure 6:**
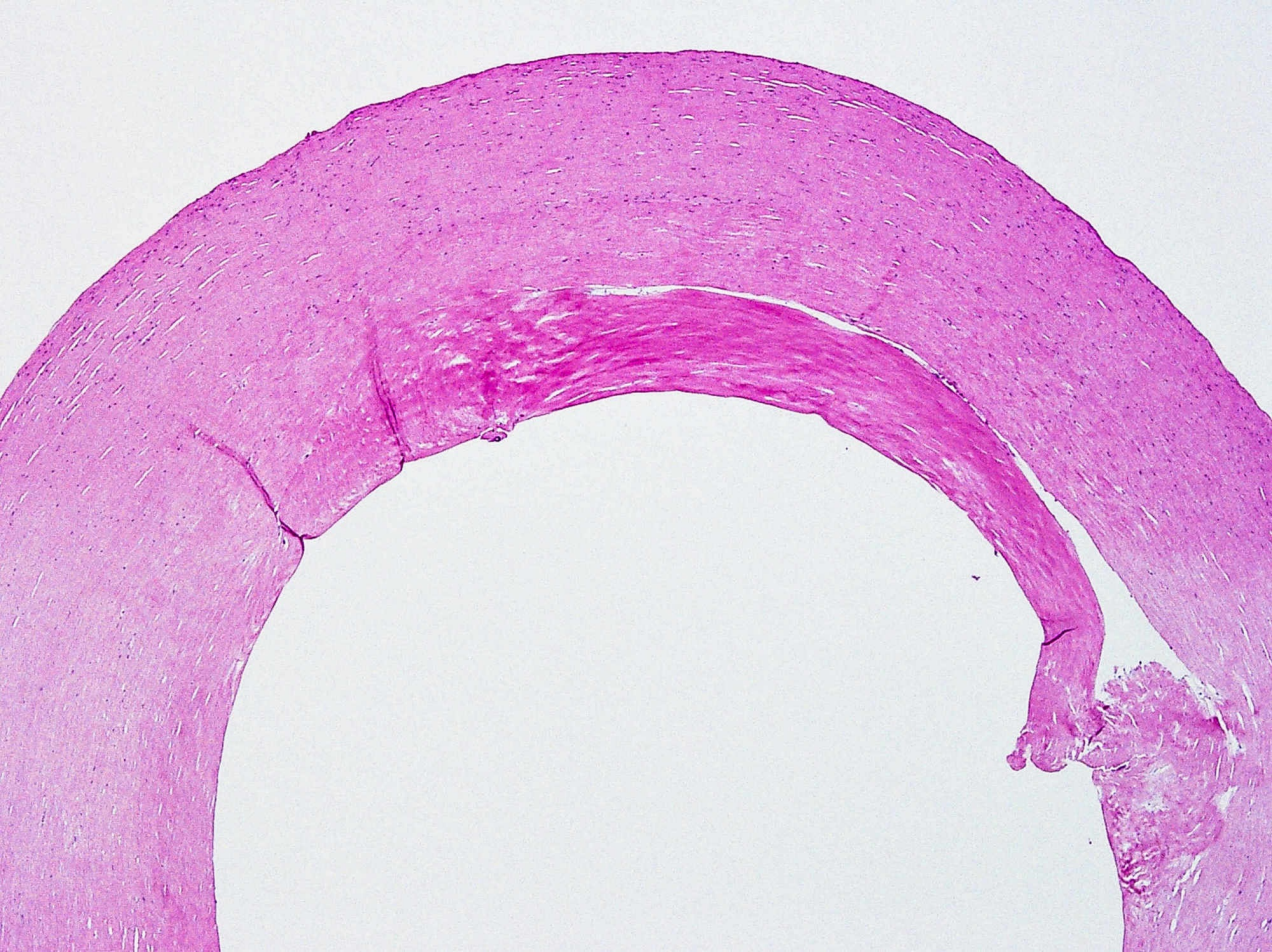
Haematoxylin and eosin stain of fibrous capsule showing mostly acellular hyalinised fibrous tissue

The patient made a full recovery and post-operative CT head demonstrated resolution of his hydrocephalus (Figure 5). Histological analysis revealed the encasing sheath to be comprised of non-inflammatory, dense, mostly acellular hyalinised fibrous tissue (Figure 6).

## Discussion

A fibrotic sheath encasing the distal tip of a peritoneal catheter in a ventriculoperitoneal shunt has previously been described in two case reports by one author [7,8]. The same author describes an additional similar case where the peritoneal catheter was encased in a fibrous capsule adherent to the peritoneal wall [7]. In contrast to our case, hydrocephalus secondary to subarachnoid haemorrhage was the indication for shunt insertion in all three of these previous reports, and one case involved a lumbo-peritoneal shunt. Additionally, in all three of these previous cases, abdominal laparoscopy was employed to remove the fibrous capsule in contrast to our case. Similar to our case, however, none of the patients in these previous reports had abdominal signs or symptoms.

A pathophysiological foreign body response to the peritoneal catheter involving mesothelial cells has been proposed to be the cause of the development of these fibrous capsules; however, this does not explain why the fibrotic capsule was limited to, and only encased the side slits at the distal end of the catheter (Figure 2) [8]. Alternatively, progressive debris accumulation in the dead space beneath the side slits may also lead to the formation of granulomatous nodules [9]. Additionally, Del Bigio et al. showed that graphite, used to coat the side slits in peritoneal catheters in order to keep them patent during storage, incites a chronic inflammatory response with subsequent deposition of granulomatous tissue which blocks the slits alone and leads to obstruction [10]. This would explain why the fibrous sheath encased only the side slits and not the remainder of the peritoneal catheter in our case.

## Conclusions

When the peritoneal catheter is suspected to be the site of obstruction in a malfunctioning ventriculoperitoneal shunt, a fibrous sheath encapsulating the distal side slits in the catheter should be suspected as a potential cause. In our case, finding a fibrous sheath obstructing an otherwise functional peritoneal catheter obviated the need to expose the entire ventriculoperitoneal shunt system, avoiding the inherent increased risk of infection when the shunt hardware is exposed. Furthermore, should such a fibrous sheath be encountered, not only should it be removed, but the catheter should also be cut short to remove the segment containing any graphite coated slits in order to prevent repeat blockage. Finally, the rationale for the continued production of peritoneal catheters with side slits should be further investigated.
